# A conserved C2H2 zinc finger protein, Rel1, links ribosome biogenesis to sexual development and antifungal susceptibility in a ubiquitous human fungal pathogen

**DOI:** 10.1128/aem.01460-25

**Published:** 2025-11-25

**Authors:** Lili Yan, Man Chen, Yuanli Liu, Pengjie Hu, Yan Wang, Yanli Cao, Ye Huang, Xiuyun Tian, Xinping Xu, Fanglin Zheng

**Affiliations:** 1 The Department of Respiratory and Critical Care Medicine, Jiangxi Provincial Key Laboratory of Respiratory Diseases, Jiangxi Institute of Respiratory Diseases, The First Affiliated Hospital, Jiangxi Medical College, Nanchang University 47861, Nanchang, Jiangxi, China; 2 Jiangxi Clinical Research Center for Respiratory Diseases, Nanchang, Jiangxi, China; 3 Jiangxi Hospital of China-Japan Friendship Hospital, Nanchang, Jiangxi, China; 4 Division of Nephrology, Renmin Hospital of Wuhan University 117921, Wuhan, China; 5 Department of Critical Care Medicine, The First Affiliated Hospital of Gannan Medical College 477808 , Ganzhou, Jiangxi, China; 6 State Key Laboratory of Mycology, Institute of Microbiology, Chinese Academy of Sciences 85387, Beijing, China; 7 College of Pharmaceutical Sciences, Southwest University 26463, Chongqing, China; 8 School of Basic Medical Sciences, Jiangxi Medical College, Nanchang University 47861, Nanchang, China; Royal Botanic Gardens Kew, Surrey, United Kingdom

**Keywords:** *Cryptococcus neoformans*, Rel1, ribosome biogenesis, sexual reproduction, antifungal susceptibility

## Abstract

Sexual development is a fundamental process that promotes environmental adaptation and the emergence of highly virulent and drug-resistant isolates in the human fungal pathogen *Cryptococcus neoformans*. Although the genetic factors involved in the sexual life cycle have been extensively investigated in this pathogenic fungus, the critical regulator(s) beyond transcriptional machinery remain poorly understood. Rei1, a conserved C2H2-type zinc finger protein, functions as a 60S ribosome biogenesis factor and plays a crucial role in maintaining cellular growth at low temperature in yeast. In this study, we identified a yeast Rei1-like protein, Rel1, based on the dynamic upregulation of its encoding gene during unisexual reproduction in *C. neoformans*. Similar to Rei1’s role in yeast, disruption of the *REL1* gene results in significant growth retardation under low temperature conditions. Consistent with its upregulation during sexual development, *REL1* deletion compromised the entire sexual cycle, including yeast-hyphae morphogenesis, basidial differentiation, meiosis, and sporulation. RNA-seq analysis revealed that Rei1 plays an important role in the transcriptional activation of key sexual reproduction genes involved in mating response, morphogenesis, and meiosis, suggesting a potential link between ribosome biogenesis and sexual development in *C. neoformans*. Furthermore, *REL1* disruption increased susceptibility to the antifungal drug 5’-flucytosine (5′-FC) but enhanced resistance to fluconazole, indicating its differential role in antifungal drug resistance mechanisms. Collectively, this study provides critical insights into how *C. neoformans* utilizes a conserved ribosome biogenesis factor to coordinate cold resistance, sexual development, and antifungal susceptibility, highlighting the crucial regulatory molecular mechanisms underlying the orchestration of these physiological processes in this ubiquitous pathogenic fungus.

**IMPORTANCE:**

*Cryptococcus neoformans*, a leading cause of fatal fungal meningitis in immunocompromised individuals, is recognized as a model organism for studying fungal sexual reproduction, as its well-characterized sexual life cycle. Sexual reproduction is a highly energy-intensive process that requires the coordination of various morphological developmental events with dynamic genomic ploidy changes to ensure successful sporulation. However, the conditions that stimulate mating are predominantly driven by nutritional limitation. How *C. neoformans* orchestrated the nutrient-poor conditions to safeguard the sequential developmental events remains elusive. In this study, we characterized a mating-responsible gene, *REL1*, which encodes a C2H2 zinc-finger protein that is highly homologous to a yeast 60S ribosome biogenesis factor Rei1. Disruption of the *REL1* gene severely impaired the entire sexual development process. Transcriptomic profiling analysis demonstrated a mating-induced upregulation of multiple Gene Ontology (GO) terms associated with ribosome biogenesis, transmembrane transport, and metabolism. Notably, it also highlighted Rei1’s requirement for activating the key sexual reproduction genes involved in mating response, morphogenesis, and meiosis/sporulation. These findings demonstrate Rel1’s multifunctionality beyond its previously characterized role in conferring cold tolerance in yeast, implicating a potential link between ribosome biogenesis, sexual development, and antifungal susceptibility in a ubiquitous human fungal pathogen.

## INTRODUCTION

*Cryptococcus neoformans* is a globally distributed human fungal pathogen and a primary cause of fatal fungal meningitis in immunocompromised individuals worldwide, accounting for approximately 180,000 deaths annually (1). Recognized as a top-ranked fungal pathogen in the newly released World Health Organization priority list, it poses an increased threat to global health (2, 3). *C. neoformans* is a heterothallic fungus with two characterized mating types: MATα and MATa. Given the well-defined sexual life cycle, it has been recognized as an ideal model organism for studying fungal sexual reproduction (4). Although it can undergo robust bisexual reproduction between two opposite mating types, unisexual reproduction within the same mating type predominates in nature due to the extremely biased distribution of the α mating type (5, 6).

Sexual reproduction plays an important role in adaptation to the environmental niches and promotes host infection by generating morphological, genotypic, and phenotypic diversity in *C. neoformans* (4). As a nonclassical dimorphic fungal pathogen, *C. neoformans* undergoes yeast-hyphae transition primarily in response to mating stimuli, a process tightly associated with sexual reproduction (7). Hyphae formation not only facilitates the expansion of its ecological niche but also enhances resistance against natural predators, such as soil amoeba (8, 9). Notably, hyphae are completely avirulent and can also confer immune protection against lethal infection by the highly virulent serotype A strain in a murine host (10–13). During sexual reproduction, meiosis drives extensive chromosome recombination, generating genetic and karyotypic diversity that contributes to the emergence of highly virulent and drug-resistant isolates (14, 15). In addition, the final products of sexual reproduction, basidiospores, are recognized as critical infection propagules due to their small size and stress-resistant feature (16, 17). Sex-specific meiotic genes are also expressed in the host during infection, which are involved in ploidy reduction of Titan cell and promoting the generation of anti-genotoxic progenies and adaptation to the host stressors (18). Given the crucial role of sexual reproduction in environmental adaptation and infection, extensive studies over the past two decades have focused on identifying the signal pathways, key transcriptional regulators, and epigenetic modifications that govern the sequential sexual development events in *C. neoformans*. Nevertheless, the regulatory mechanisms beyond transcriptional machinery remain poorly understood.

Genome-wide protein synthesis depends on the efficient translation of newly synthesized mRNA, which is crucial for responding to biotic/abiotic stress and achieving environmental adaptation. As a protein synthesis machinery, the accurate and efficient translation of transcripts is ensured by the precise assembly of functionally active 40S and 60S ribosomal subunits with tRNAs to form mature 80S ribosomes. Pre-ribosome particles are initially assembled in the nucleolus, subsequently processed in the nucleoplasm, and finally exported to the cytoplasm for final maturation (19). During the assembly of the pre-ribosome particle, various ribosome biogenesis factors are engaged and incorporated into the particle to ensure the sequential ribosome maturation. Among these, Rei1 (required for isotropic bud growth 1) is a well-characterized factor that plays a critical role in the late-stage maturation of the 60S subunit (20). Rei1 is a conserved C2H2-type zinc finger protein that was first identified as an interacting partner of the septin-binding protein Nis1 in the model yeast *Saccharomyces cerevisiae* (21). This discovery unveiled that Rei1 potentially functions as a component of the mitotic signaling network and negatively regulates Swe1 kinase activity (21). Rei1 localizes to the cytoplasm throughout the cell cycle, and disruption of its encoding gene results in a severe growth defect under cold stress conditions, indicating its critical role in maintaining cell growth at low temperatures in yeast. Further studies demonstrated that Rei1 is required for the nuclear recycling of a pre-60S ribosomal subunit-associated factor, Arx1, a methionine aminopeptidase involved in assembly of pre-ribosomal particles during the biogenesis of the 60S ribosomal subunit (22). In addition, Rei1 plays a crucial role in coordinating the dissociation and recycling of at least three nucleocytoplasmic pre-60S factors from the late cytoplasmic pre-60S particle into the nucleus (23). In the absence of Rei1, the nuclear import of Arx1, Alb1, and Tif6 is blocked, preventing formation of the large ribosome subunit and defects in the nuclear processing of the pre-60S subunit (23). Structural studies revealed that the C-terminal segment of Rei1 is deeply inserted into the polypeptide tunnel of the pre-60S particle, which may function as a quality check for the tunnel integrity and requirement for the subsequent steps of 60S maturation (24). Intriguingly, except for its well-defined role in the maturation of pre-60S ribosome subunit, multiple genetic studies also revealed a conserved function of Rei1 and Rei1-like proteins in achieving cold resistance in yeast and plants. Reh1, a Rei1 homolog in *S. cerevisiae,* functions redundantly with Rei1 in the cytoplasmic maturation of the 60S subunit (25). Overexpression of *REH1* can partially restore the observed cold-sensitive phenotype in the *rei1*Δ mutant (21, 23). Similarly, in *Arabidopsis thaliana*, the Rei1-like proteins REIL1 and REIL2 are required for leaf growth under cold stress conditions but not for growth at optimal temperature (26). Notably, in the entomopathogenic fungus *Beauveria bassiana*, BbRel1 (the yeast Rei1 homolog) is not only required for maintaining cell growth at low temperatures but also plays a crucial role in controlling the expression of genes involved in nutritional metabolism essential for the asexual cycle *in vitro* and its life cycle within the host (27). These results revealed a pleiotropic role of Rei1-like proteins that is beyond their involvement in maintaining cell growth under low temperatures in filamentous fungi.

In this research, we identified a Rei1 ortholog, Rel1, in the globally prevalent basidiomycetous pathogenic fungus *C. neoformans*, based on its significantly upregulated expression in response to mating stimuli. Consistent with the role of ScRei1 in *S. cerevisiae*, the disruption of *REL1* resulted in a marked cold-sensitive growth phenotype, suggesting a conserved role in maintaining cellular growth at cold temperatures across diverse species. Consistent with its upregulated expression in response to mating induction, *REL1* deletion led to a severe defect in sexual development. Furthermore, Rel1 was differentially involved in anti-fungal drug susceptibility, as its deletion caused increased sensitivity to 5-flucytosine but exhibited significantly enhanced resistance to fluconazole. Collectively, our findings demonstrate a pleiotropic function of Rel1 in *C. neoformans*, extending our understanding of this well-defined ribosome biogenesis factor in orchestrating various physiological processes in this global human fungal pathogen.

## RESULTS

### Identification of *REL1*, a Rei1-like protein-encoding gene with dynamically mating-induced upregulation in *C. neoformans*

**Fig 1:**
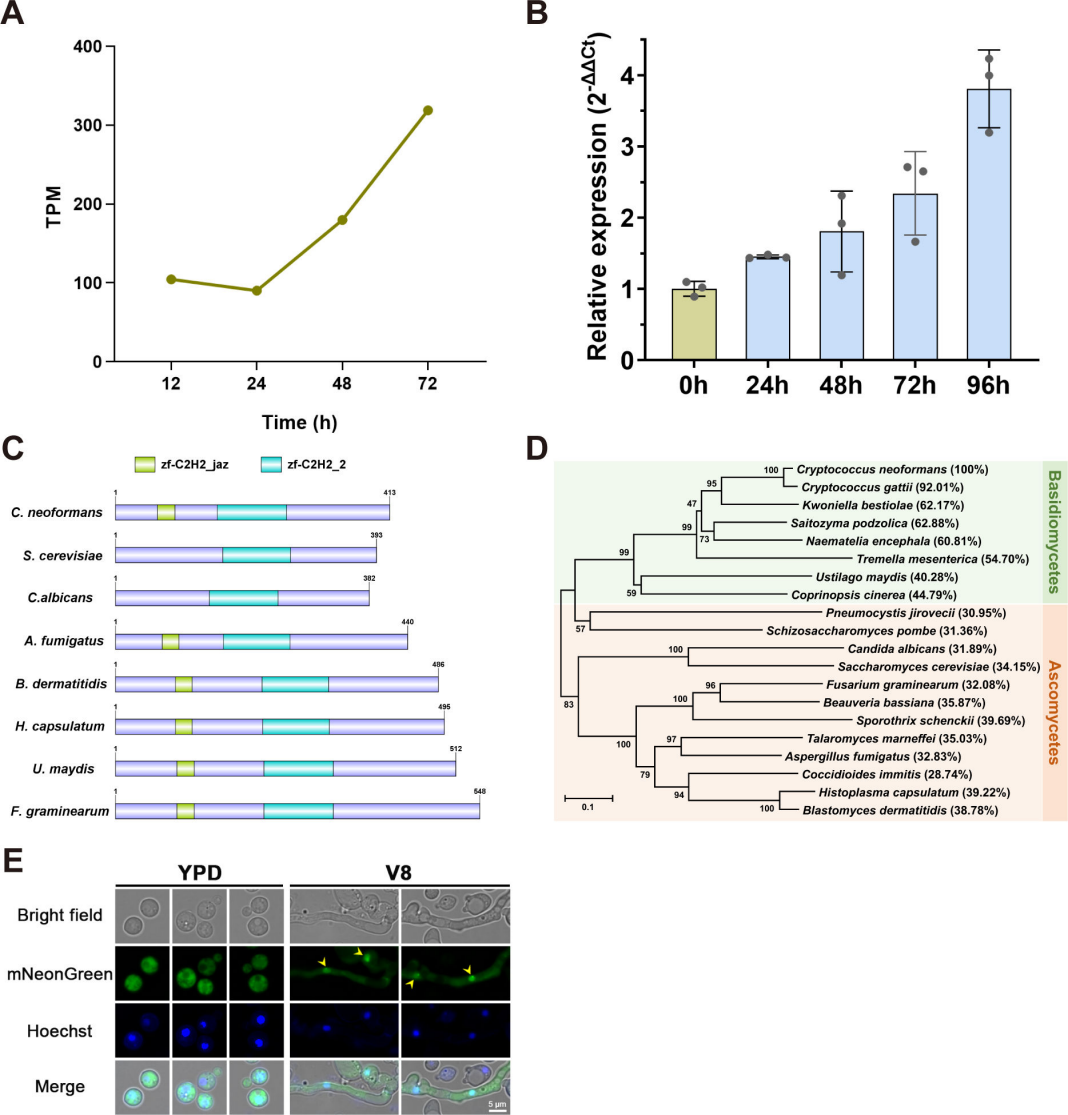
Identification of a conserved C2H2 zinc finger protein, Rel1, that is upregulated in response to mating stimulation. (**A**) Transcriptional dynamics of the *REL1* gene during unisexual development from publicly available RNA-seq data (28). TPM refers to transcripts per million. (**B**) Time course RT-qPCR assay revealed gradually increased expression of *REL1* in response to mating stimulation. Error bar indicated mean ± SD from three biological replicates. (**C**) Diagrams of domain organization of Rel1 orthologs from ascomycetes and basidiomycetes. The fungal species exhibited in the diagram include *C. neoformans*, *S. cerevisiae*, *C. albicans*, *Aspergillus fumigatus*, *Blastomyces dermatitidis*, *Histoplasma capsulatum*, *Ustilago maydis*, and *Fusarium graminearum*. (**D**) Phylogenetic tree displays Rel1 orthologs from representative fungal species within ascomycetes and basidiomycetes. Sequence alignments were performed using ClustalW, and the neighbor-joining tree was generated using MEGA 7 software. The Poisson model was employed as the substitution model for neighbor-joining phylogenetic analysis. Numbers on the tree branches represent the bootstrap support calculated per 1,000 bootstrap replicates. Sequence identity between the Rel1 protein of *C. neoformans* and its homologs from other fungal species is indicated within the brackets. (**E**) Subcellular localization of mNeonGreen-Rel1 fusion protein under mating repressing (YPD) and inducing (V8) conditions. Yellow arrows indicate the nuclear-localized signal of mNG-Rel1 on V8 medium, which colocalized with the Hoechst-stained nucleus signal. Scale bar, 5 µm.

To identify the potential regulators of yeast-hyphae morphogenesis and sexual reproduction in *C. neoformans var. neoformans*, we focused on a C2H2 zinc finger protein-encoding gene, *CNB00600,* which was dynamically upregulated in response to mating stimuli according to publicly available RNA-seq data (28) (Fig. 1A). Time-course RT-qPCR confirmed the transcriptional upregulation of this gene during the unisexual cycle in the serotype D reference strain XL280 cultured on V8 medium (Fig. 1B). *CNB00600* gene harbors a 1418 bp open reading frame (ORF) with three introns, encoding a protein composed of 413 amino acids with a predicted molecular weight of 46.4 kDa. Structural domain analysis performed with the NCBI CDD program revealed two C2H2 zinc finger domains within this protein: a central zf_C2H2_2 zinc finger domain (156–252 aa, E-value = 1.10e–44) and a N-terminal zf_C2H2_jaz zinc finger domain (67–90 aa, E-value = 2.04e–07) (Fig. 1C). Phylogenetic analysis showed that its orthologs were widely distributed across Ascomycetes and Basidiomycetes, including various human and plant fungal pathogens as well as model fungal species, with similar domain architectures (Fig. 1C and D). Further sequence alignment showed 34.15% sequence identity and 48.15% similarity to a C2H2 zinc finger protein Rei1, a well-characterized 60s ribosome biogenesis factor involved in the maturation of the pre-60S ribosome in *S. cerevisiae* (24, 25). We thus named this conserved zinc finger protein Rel1 (Rei1-like protein 1) based on its homology to ScRei1. In contrast to a unique central C2H2 zinc finger domain present in *Saccharomyces cerevisiae* and *Candida albicans* (order Saccharomycetales), most Rel1 orthologs from other orders exhibit two C2H2 zinc finger domains (Fig. 1D), suggesting divergent structural evolution among fungi.

As the *REL1* gene is upregulated in response to mating stimuli, we investigated the subcellular localization of Rel1 under vegetative growth and sexual reproduction conditions. To this end, recombinant strains with an N-terminal mNeonGreen fluorescent-tagged Rel1 under the control of either its native promoter or the copper-responsive promoter P*_CTR4_* were constructed, respectively. However, the native promoter-driven expression of mNeonGreen-Rel1 is barely detectable under fluorescence microscope under both mating repressing (YPD) and mating inducing (V8) conditions (data not shown), indicating a low basal expression level of this gene. In contrast, fluorescence signal was clearly observed in the recombinant strain expressing mNeonGreen-Rel1 under control of the *CTR4* promoter (Fig. 1E). Under mating repressing conditions (YPD), mNeonGreen-Rel1 exhibited a diffused localization in the cytoplasm in the yeast cells. When shifted to mating-inducing condition (V8), both cytoplasmic and nuclear localization signals were observed within the hyphae cells (Fig. 1E). This result suggested a cytoplasmic-nuclear shuttling of Rel1 upon shifting from the mating-repressing condition to the mating-inducing condition, implying its potential role in regulating mating and sexual reproduction in *C. neoformans*.

### Rel1 plays a critical role in cold tolerance, cell cycle progression, and cell size maintenance in *C. neoformans*

**Fig 2:**
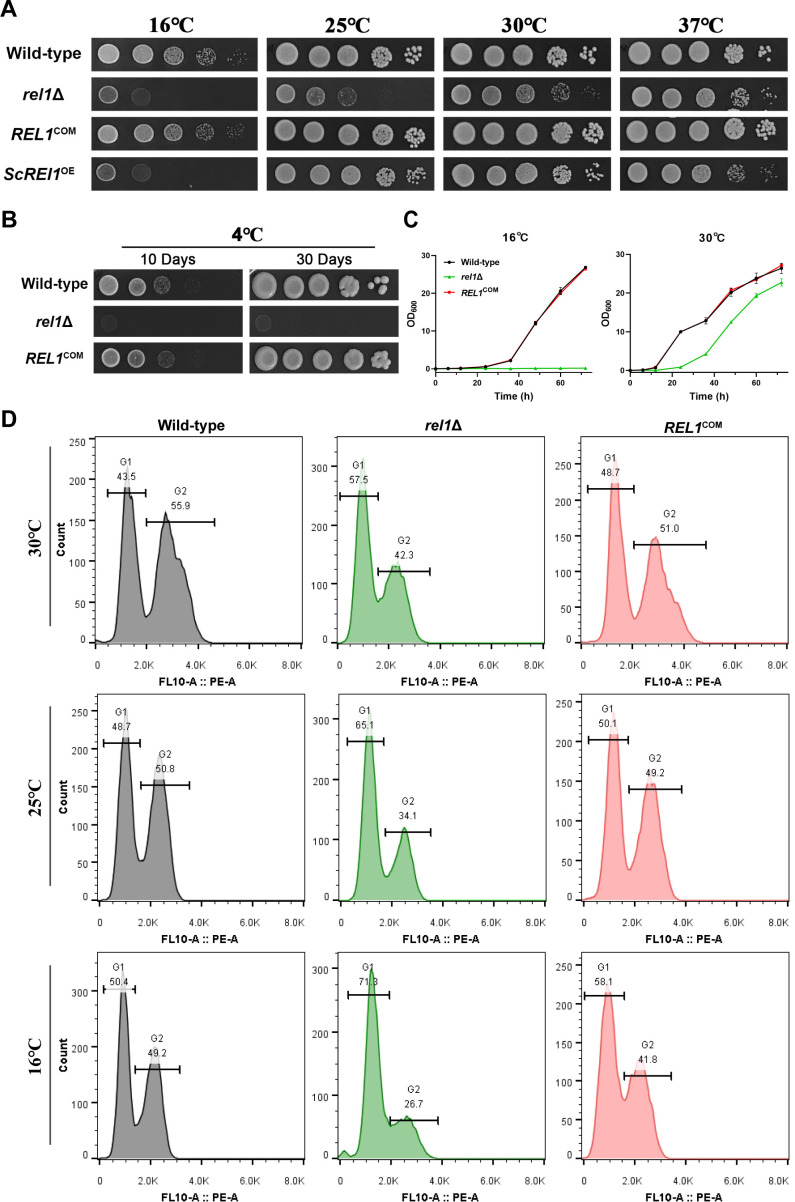
Rel1 plays a critical role in maintaining cellular growth and cell cycle distribution at low temperatures in *C. neoformans*. (**A**) Spotting assay of *rel1* deletion, complementation, and wild-type strains on YPD agar medium at different temperatures. Ten times the indicated strains were diluted for spotting, and images were captured after 3 days of incubation at 37°C, 30°C, 25°C, and 16°C, respectively. (**B**) Extreme cold temperature tolerance analysis of the *rel1* deletion, complementation, and wild-type strains at 4°C. Images were captured after 10 and 30 days of incubation, respectively. (**C**) Growth curve assay of the indicated strains on YPD liquid medium at 30°C and 16°C, respectively. (**D**) Cell cycle assay of the WT, *rel1* deletion, and complementation strains cultured at the indicated temperatures.

To explore the genetic function of Rel1 in *C. neoformans var. neoformans*, a *rel1* deletion mutant was generated in the serotype D reference strain XL280, which was widely used as a model strain for studying fungal sexual reproduction (4, 29). As ScRei1 and Rei1-like proteins play a crucial role in maintaining cellular growth at low temperatures in yeast and plants (21, 26), we initially examined the impact of Rel1 on cryptococcal growth across a temperature range of 16°C to 37°C. As expected, deletion of the *REL1* gene resulted in significant growth retardation after 3 days of incubation on YPD agar medium at 25°C and much more dramatic inhibition of growth at a lower temperature (16°C) (Fig. 2A). In contrast, only marginal reduction or comparable level of growth was observed at 30°C and 37°C compared with the wild-type strains, respectively. After 1 week of incubation at 16°C, the growth of the *rel1*Δ mutant gradually recovered (data not shown), indicating a growth inhibition rather than an adaptation defect of this mutant at this low temperature. Complementation with a wild-type copy of the *REL1* gene completely restored the cold-sensitive growth phenotype observed in the *rel1*Δ mutant (Fig. 2A). Notably, after an extended incubation to 30 days at a lower temperature of 4°C, the *rel1*Δ mutant displayed almost negligible growth compared with both the wild-type and complemental strains (Fig. 2B). These observations suggested that Rel1 is indispensable for achieving cellular growth under low temperatures in *C. neoformans*, indicating a conserved role of Rei-like proteins in conferring cold tolerance among Ascomycetes and Basidiomycetes. Next, the expression dynamics and subcellular localization of Rel1 in response to low temperature stimulation were investigated. RT-qPCR analysis revealed that the transcription level of *REL1* was significantly downregulated following temperature shifts from 30°C to 25°C and 16°C (Fig. S1A). The contradiction between the crucial role of *REL1* in conferring cold tolerance and its downregulation upon exposure to low temperature implies that maintaining a relatively low basal expression level of *REL1* may represent an important adaptive strategy in response to cold stress. Consistent with its putative role as a late maturation factor of the 60S ribosomal subunit, a process that exclusively occurs in the cytoplasm (20, 23, 30), mNeonGreen-Rel1 was consistently localized in the cytosol under these tested culture conditions, including 30°C and lower temperatures (25°C and 16°C) (Fig. S1B). To further test functional conservation of Rei1 and Rei1-like proteins, we introduced *S. cerevisiae* Rei1 (YBR267W) encoding genes into the *rel1*Δ strain under control of the *CTR4* promoter. Growth assay under different temperatures revealed that complementation with ScRei1 completely restored growth retardation at 25°C (Fig. 2A). However, the failure of ScRei1 complementation in compensating for the growth defect at 16°C also suggests a diverse function between these proteins.

The significant growth delay observed in the *rel1*Δ mutant at low temperatures implied potential cell cycle arrest during either the G1, G1/S transition, or G2 phase, as ScRei1 had been implicated in the mitotic signaling network in *S. cerevisiae* (21). We then investigated whether Rel1 was involved in the cell cycle progression in *C. neoformans*. To this end, the cell cycle of the wild-type, the *rel1* disruption, and complemented strains was assessed via flow cytometry under different temperature growth conditions. This assay revealed that *rel1* disruption resulted in a reduced G2 phase population and an increased ratio of G1 and G1/S phase cells compared with that in the wild-type strain at 30°C, whereas the complemented strain restored the G1/G2 ratio to wild-type strain level (Fig. 2D). As the temperature drops (at 25°C and 16°C), a more dramatic reduction in G2 population cells and a higher G1/G2 ratio were observed in the *rel1*Δ mutant (Fig. 2D). These results indicated a potential G1 phase or G1/S transition arrest in the *rel1*Δ mutant in response to cold stress. Taken together, these results demonstrated a critical role of Rel1 in maintaining cellular growth and proper cell cycle progression under cold stress conditions in *C. neoformans*.

Next, we further examined the impact of *REL1* disruption on cellular morphology and cell size under various culture temperatures, as ribosome activity is tightly associated with cell size (31, 32). As shown in Fig. S2A, the *rel1*Δ mutant, exhibited typical yeast morphology across these tested culture temperatures, which is similar to the wild-type and the *REL1* complement (*REL1*^COM^) strain. However, a noticeable reduction in cell size was observed in the *rel1*Δ mutant compared with that observed in WT and *REL1*^COM^ strains under all tested temperature conditions (Fig. S2A). Quantitative analysis confirmed that the diameter of the cell population of the *rel1*Δ mutant was significantly smaller than that of the wild-type strain at both 30°C and lower temperatures, whereas the complementation of the *REL1* gene fully restored the cell size to the wild-type level (Fig. S2B). These results indicate that Rel1 plays an important role in maintaining normal cell size across different culture temperatures, potentially due to its involvement in ribosome biogenesis.

### Rel1 plays an important role in yeast-hyphae morphogenesis and sexual reproduction during the cryptococcal sexual cycle

**Fig 3:**
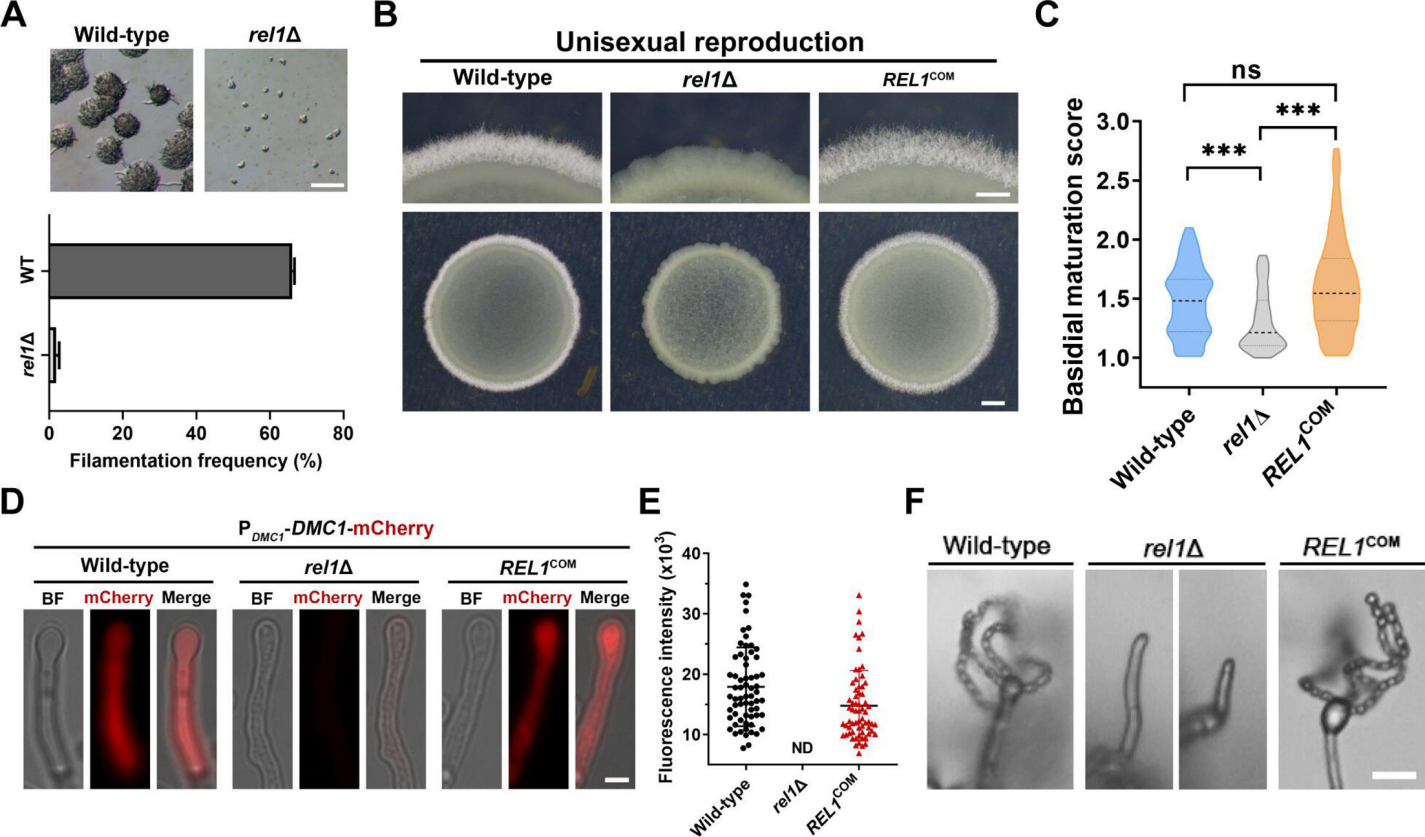
Rel1 played an important role in facilitating sexual reproduction. (**A**) Filamentation initiation assay of the WT and *rel1*Δ strains under unisexual mating conditions. The mini-colonies of the indicated strains were photographed after 24 h of inoculation on V8 agar medium at 25°C in the dark. Scale bar, 100  µm. (**B**) The effect of *REL1* deletion and complementation on hyphal development at the colony level during unisexual reproduction. Images were captured under a stereoscope after 5 days of incubation on V8 agar medium. Scale bars represent 200 µm for the upper panel and 1 mm for the lower panel, respectively. (**C**) Basidial maturation score assessment of the hyphae population from the WT, *REL1* deletion, and complemental strains. Eighty basidia of each strain were calculated at the 7th day of inoculation on V8 medium. ***, *P* < 0.001; ns, not significant (two-tailed Student’s *t*-test). (**D**) Fluorescent observation of the Dmc1-mCherry expression signal in wild-type, *rel1* disruption, and complementation strain background. Scale bar, 5  µm. (**E**) Quantitative assay of Dmc1-mCherry fluorescent intensity from the indicated strains after 7 days of incubation on V8 medium. Fluorescent signals of 65 basidia of each strain were calculated. (**F**) The impact of Rel1 on sporulation during unisexual reproduction. The images of hyphae tips with or without a spore chain were captured after 3 weeks of incubation on V8 medium. Scale bar, 10  µm.

Given the significant upregulation of *REL1* in response to mating stimulation on V8 medium (Fig. 1A and B), we investigated its involvement in sexual development in *C. neoformans*. As shown in Fig. 3A, the *rel1*Δ mutant exhibited a markedly impaired unisexual filamentation initiation compared with the wild-type strain at the early mating stage (24 h). Consistently, colony-level filamentation observation showed that few hyphae were generated from the *rel1*Δ mutant after 3 days of incubation on V8 medium, whereas robust filamentation was observed in both wild-type and the complemented strain (Fig. 3B). After extended incubation on V8 for more than a week, only sporadic hyphae could be observed at the colony edge of the *rel1*Δ mutant. These findings indicated a crucial role of Rel1 in facilitating the yeast-hyphae transition and hyphal development during cryptococcal unisexual reproduction.

As yeast-hyphae morphogenesis is tightly coordinated with sexual reproduction, we then examined the impact of Rel1 in late sexual development processes, including basidial maturation, meiosis, and sporulation. To quantitatively investigate the impact of Rel1 on basidial differentiation, a previously developed basidial maturation score (BMS) analysis method was applied (28). As shown in Fig. 3C, a dramatically reduced BMS level of the sporadic hyphae generated from the *rel1*Δ mutant was observed, compared with those detected from wild-type and the complemental strain, indicating a critical role of Rel1 in achieving basidium differentiation and maturation during sexual development. Previous research revealed that basidial maturation and meiosis were spatiotemporally coordinated during sexual reproduction to ensure successful basidiospore production (28). We then addressed whether Rel1 was required for meiosis by investigating the expression of the meiosis-specific recombinase Dmc1, a molecular marker of meiosis expressed exclusively within the basidium (7). To this end, a P*_DMC1_*-DMC1-mCherry reporter construct was transformed into *REL1* deletion, complemented strains, and wild-type strains, respectively. The Dmc1-mCherry fluorescent signal was then monitored during the late stage of sexual development. As shown in Fig. 3D, *rel1* disruption resulted in barely detectable Dmc1-mCherry expression, whereas strong signals were observed in the hyphae tips from the complemented strain and wild-type background. These results indicated that Rel1 played a critical role in the activation of meiosis during cryptococcal unisexual reproduction. Subsequently, we examined Rel1’s role in the generation of the final sexual reproduction product, basidiospore. As shown in Fig. 3F, after 2 weeks of cultivation on V8 medium, substantial basidiospores and spore chains were generated in the WT and the complemented strain but were completely absent in the *rel1* deletion mutant. These results revealed a crucial role of Rel1 in facilitating cryptococcal unisexual reproduction.

To determine whether Rel1 is involved in bisexual reproduction, a *rel1*Δ**a** strain was generated in the XL280**a** background, and a bilateral bisexual mating assay was performed by setting up crosses between the two opposite mating type strains of the *rel1*Δ mutant (*rel1*Δα × *rel1*Δ**a**) and wild-type mutant (XL280α × XL280 **a**). Similar to the phenotype observed during unisexual mating, only sparse hyphal growth was exhibited in the bisexual mating colony of the *rel1*Δ mutants after 5 days of incubation on V8 medium, whereas the wild-type bisexual mating colony displayed robust filamentation (Fig. S3A). After extending the incubation to 2 weeks, typical spore chains were generated from the wild-type bisexual mating, whereas a complete loss of sporulation was observed from the bisexual mating of the *rel1*Δ mutants (Fig. S3B). Collectively, these results indicate that Rel1 is indispensable for the completion of both unisexual and bisexual cycles in *C. neoformans*.

### Rel1 regulates cryptococcal sexual reproduction by facilitating the expression of genes involved in mating and sexual development

To deeply investigate the regulatory function of Rel1 on sexual reproduction, we performed RNA-seq analysis to examine the genome-wide transcriptional profiles of wild-type and *rel1*Δ strains cultured on mating-inducing V8 medium or mating-suppressing YPD medium. Principal component analysis (PCA) demonstrated that the three biological replicates of each sample clustered tightly together (Fig. S4A), indicating high reproducibility and consistency of the RNA-seq data. Analysis of the RNA-seq data revealed that 2,427 genes were differentially expressed (threshold │log2(Fold change)│> 1.0, *q*-value <0.05) in the wild-type strain on mating-inducing V8 medium compared with mating-repressing medium (YPD), including 1,511 significantly upregulated genes and 916 significantly downregulated genes (Fig. S4B and Table S1). The substantial number of differentially expressed genes indicated significant transcriptional reprogramming in response to mating stimulation, as examined in a previous RNA-seq analysis of the wild-type strain under identical conditions (33). Similarly, 2,710 genes were differentially expressed in the *rel1*Δ strain compared with WT under mating-inducing conditions, including 1,559 downregulated genes and 1,151 significantly upregulated genes (Fig. S4B and Table S2). Since Rel1 played a positive role in regulating sexual development, we focused on the gene sets that were downregulated in the *rel1*Δ mutant compared with the WT on V8 medium (downregulated genes in the *rel1*Δ-vs-WT_V8 group) and upregulated in the wild-type strain in response to mating induction (upregulated genes of the WT_V8-vs-WT_YPD group).

**Fig 4:**
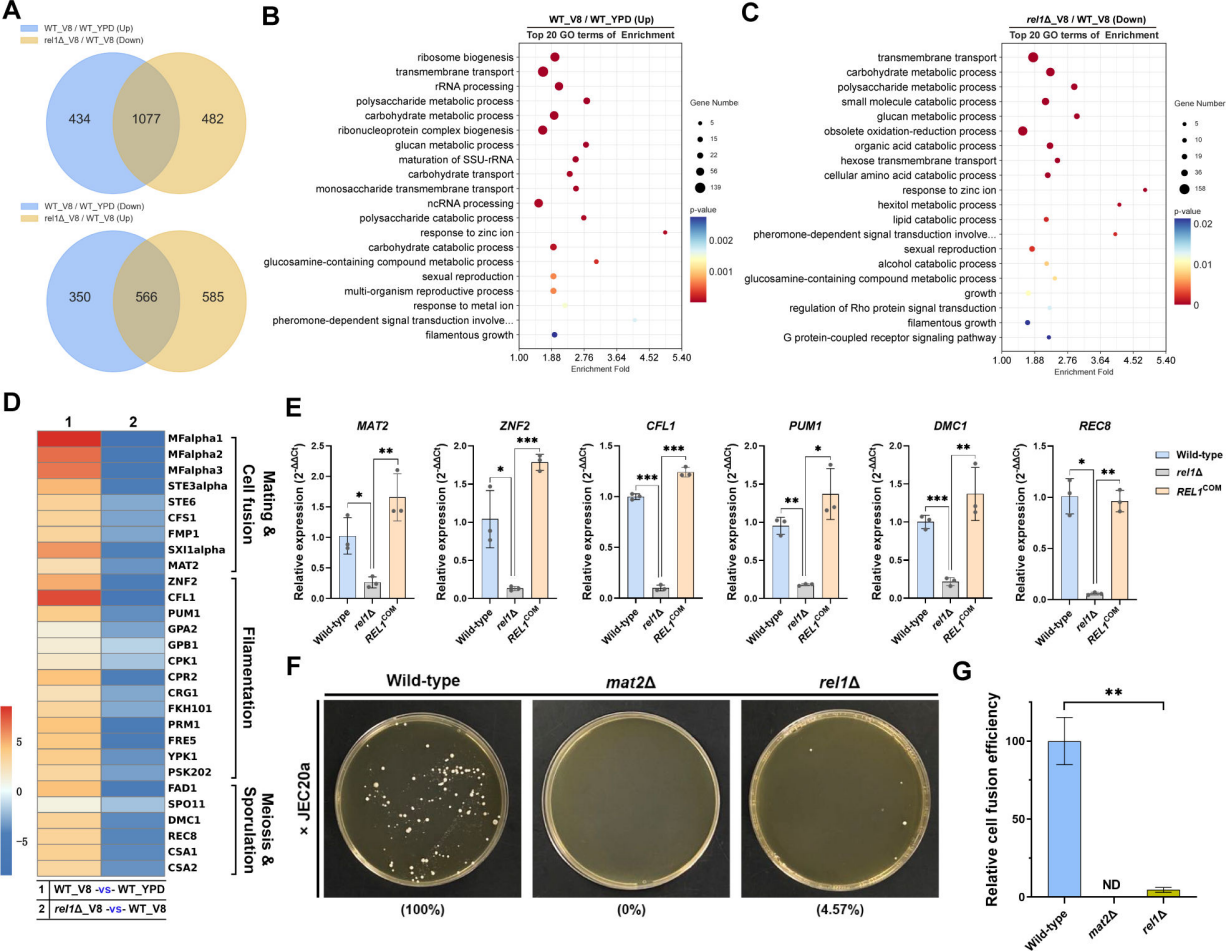
RNA-seq analysis revealed Rel1’s role in facilitating sexual reproduction. (**A**) Venn diagrams of the differentially expressed genes of the WT_V8/WT_YPD group and *rel1*Δ_V8/WT_V8 group. (B, C) The top 20 enriched Gene Ontology (GO) terms among the upregulated genes in the WT_V8/WT_YPD group (**B**) and the downregulated genes in the *rel1*Δ_V8/WT_V8 group (**C**) are presented. (**D**) Heatmap analysis of the genes involved in mating/cell fusion, filamentation, and meiosis/sporulation in the WT_V8/WT_YPD and *rel1*Δ_V8/WT_V8 groups. Log2(fold change) of each gene derived from three biological replicates in the indicated groups is presented. (**E**) RT-qPCR assay of the key sexual development genes, *ZNF2*, *CFL1*, *PUM1,* and *DMC1*, in WT, *rel1* deletion, and complemented strains under mating-inducing conditions. Error bar indicates mean ± SD from three independent biological replicates. Statistical significance was determined by a two-tailed Student’s *t*-test (***, *P* < 0.001; **, *P* < 0.01; *, *P* < 0.05). (**F**) Cell-cell fusion assay of the indicated crosses on the double-drug selective plate after 14 h of incubation on V8 medium at 25°C in the dark. The mean relative cell fusion efficiency from three biological replicates of each cross is labeled at the bottom. (**G**) Quantification of the cell fusion efficiency of the indicated crosses from panel (**F**). Error bars represented mean ± SD from three biological replicates. Statistically significant differences are indicated by the asterisks (**, *P* < 0.01, two-tailed Student’s *t*-test). ND, not detected.

Comparative transcriptome analysis revealed that 1,077 of the upregulated genes in the wilt-type strain in response to mating stimulation (WT_V8-vs-WT_YPD group) were downregulated in the *rel1*Δ mutant (*rel1*Δ_V8-vs-WT_V8 group) (Fig. 4A), accounting for 71.3% of the upregulated genes in the WT_V8-vs-WT_YPD group and 69.1% of the downregulated genes in the *rel1*Δ_V8-vs-WT_V8 group. Conversely, 566 downregulated genes in the WT_V8-vs-WT_YPD group were upregulated in the *rel1*Δ-vs-WT_V8 group (Fig. 4A). This result indicated a critical role of Rel1 in achieving the transcriptional reprogramming in response to mating stimulation. Gene Ontology (GO) enrichment analysis of the upregulated genes in the WT_V8-vs-WT_YPD group revealed significant enrichment in multiple biological processes, including ribosome biogenesis (GO:0042254), transmembrane transport (GO:0055085), carbohydrate metabolism (GO:0005975), sexual reproduction (GO:0019953), and pheromone-dependent signal transduction involved in conjugation with cellular fusion (GO:0000750) (Fig. 4B). The significant enrichment of GO terms related to sexual reproduction and pheromone-dependent signal transduction matched well with the cellular response under mating-inducing condition. Notably, the ribosome biogenesis process emerged as the most significantly enriched GO term within this group (Fig. 4B, enrichment fold = 1.97, *P*-value = 1.14E-11), indicating a strong association between this fundamental biological process and sexual development in *C. neoformans*. Consistent with the putative role of Rel1 in 60S ribosome maturation, its encoding gene was identified in the upregulated gene list within this highly enriched GO term “ribosome biogenesis” in the WT strain in response to mating stimulation (WT_V8-vs-WT_YPD group), implying the involvement of Rel1 in ribosome biogenesis and sexual development. Conversely, GO enrichment analysis of the *rel1*Δ-vs-WT_V8 group demonstrated that many of these upregulated biological processes in the wild-type strain in response to mating induction were downregulated once *REL1* was disrupted. These processes included transmembrane transport (GO:0055085), carbohydrate metabolism (GO:0005975), cellular amino acid metabolism (GO:0009063), sexual reproduction (GO:0019953), and pheromone-dependent signal transduction involved in conjugation with cellular fusion (GO:0000750) (Fig. 4C). Heatmap analysis revealed that the mating-induced expression of the core gene sets involved in the entire cryptococcal sexual development, including mating/cell fusion, filamentation, meiosis, and sporulation, was dramatically downregulated in the *rel1*Δ mutant (Fig. 4D). This genome-wide transcriptional profile analysis demonstrated a critical role of Rel1 in facilitating the activation of cryptococcal mating and sexual reproduction.

To further confirm the remarkably downregulated expression of sexual development genes in the *rel1*Δ mutant observed from RNA-seq data, RT-qPCR assay was performed in wild-type, *rel1* disruption, and complemental strains under mating-inducing conditions. In accordance with the RNA-seq results, transcription levels of the key genes involved in mating (*MAT2*), hyphal development (*ZNF2*, *CFL1,* and *PUM1*), and meiosis/sporulation (*DMC1*) were dramatically reduced in the *rel1*Δ mutant compared with that from wild-type (Fig. 4E). In contrast, complementation with the *REL1* gene completely restored the expression of these genes to wild-type level (Fig. 4E). Among these downregulated sexual reproduction genes, *MAT2* is a master transcription factor that governs mating response processes, including pheromone production, sensing, and cell fusion, which was the prerequisite for the subsequent sexual development events. The remarkably decreased expression of *MAT2* in the *rel1*Δ mutant implied that the mating response may be impaired in the *rel1*Δ mutant. To confirm this possibility, a bisexual mating-mediated cell-cell fusion assay was performed by applying wild-type (*MATα*, SH3::NEO), *rel1*Δ (*MATα*, *rel1*::NEO), and *mat2*Δ (*MATα*, *mat2*::NAT) strains crossed with HYG marker labeled JEC20**a** strain (*MATa*, SH3::HYG). As expected, no cell fusion products were observed in the cross with *mat2*Δ mutant, indicating the dominant role of Mat2 in mediating mating response and cell fusion. Similarly, disruption of *rel1* also dramatically compromised cell fusion (4.57% ± 1.58%) compared with that in the wild-type strain (Fig. 4F and G). Taken together, these results indicated that Rel1 played a crucial role in activating mating response in *C. neoformans*.

### Rel1 plays an important role in environmental stress response and antifungal drug susceptibility

**Fig 5:**
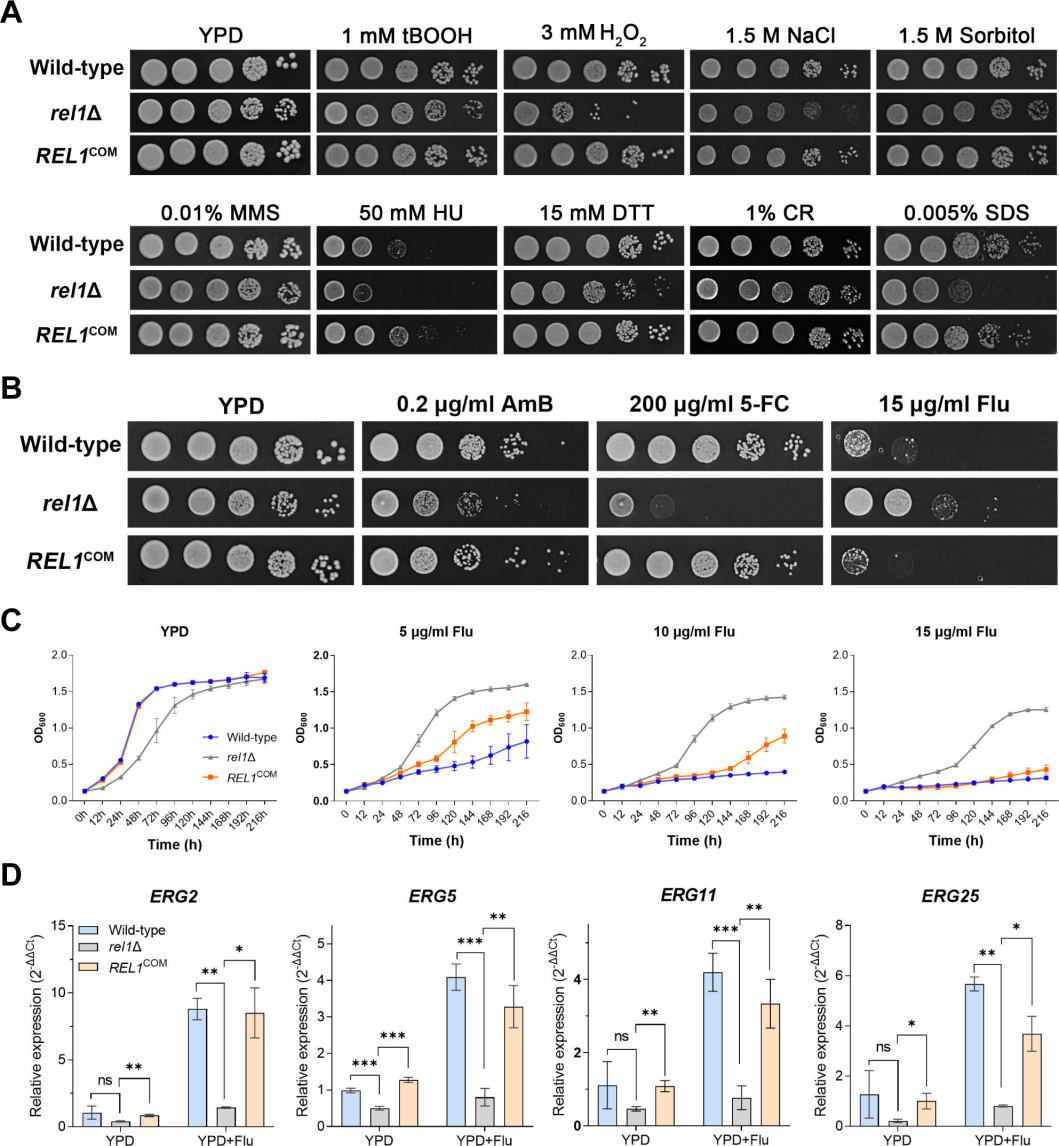
Rel1 plays a critical role in maintaining cell membrane integrity and is differentially involved in modulating antifungal drug susceptibility. (**A**) Spotting assay of *rel1* deletion, complementation, and wild-type strains under the indicated stress conditions. Images were captured after 3 days of incubation at 30°C. (**B**) Antifungal susceptibility assay of the *rel1* deletion, complementation, and wild-type strains on YPD medium containing 0.2 µg/mL amphotericin B, 200 µg/mL 5’-flucytosine, and 15 µg/mL fluconazole. Plates were incubated at 30°C for 3 days prior to photographing. (**C**) Growth curve assay of *rel1* deletion, complementation, and wild-type strains on liquid YPD and YPD supplied with different concentrations of fluconazole. (**D**) RT-qPCR analysis of the four well-defined ergosterol biogenesis genes on YPD and YPD supplied with 15 µg/mL fluconazole. Error bars represented mean ± SD from three biological replicates. Statistically significant differences are indicated by the asterisks (*, *P* < 0.5; **, *P* < 0.01; ***, *P* < 0.001; ns, not significant. Two-tailed Student’s *t*-test).

The ability of *C. neoformans* to adapt to biotic and abiotic stressors represents a fundamental attribute that enables this fungal pathogen to surmount environmental challenges and successfully establish infections in hosts. To systematically investigate Rel1’s role in adapting and/or resisting to environmental stressors, we conducted comprehensive phenotypic profiling of the *rel1*Δ mutant under various stress conditions, including oxidative stress (H_2_O_2_ and tBOOH), osmotic stress (NaCl and Sorbitol), genotoxic stress (MMS and HU), endoplasmic reticulum stress (DTT), and cell wall/membrane destabilizing stress (Congo red and SDS), respectively. As shown in Fig. 5A, after 3 days of incubation, the *rel1*Δ mutant exhibited a markedly reduced growth on H_2_O_2_, hydroxyurea (HU), and sodium dodecyl sulfate (SDS)-containing medium. *REL1* complementation fully restored growth under these conditions. In contrast, its resistance to other stressors was largely comparable with that of the wild-type. These results indicated an important role of Rel1 in conferring resistance to oxidative and genotoxic stress and maintaining cell membrane integrity.

Next, we examined whether Rel1 is involved in adapting to nutrition-limited conditions. To this end, the growth assay was performed by spotting the *REL1* deletion, complementation, and wild-type strains onto carbon starvation medium (YP without glucose or any other carbon source) and nitrogen limitation medium (SD-LN medium containing 0.05% (NH_4_)_2_SO_4_). As shown in Fig. S4A, the *rel1*Δ mutant, displayed a comparable growth with that of the WT and *REL1*^COM^ strains on these nutrition-limited media, indicating that Rel1 plays a minimal role in growth under C/N nutrient-limited conditions. To further investigate the involvement of Rel1 in adapting to host-relevant culture conditions, we investigated the survival rate of the *rel1*Δ mutant on RPMI medium with 5% CO_2_ and incubation at host-mimicking temperature 37°C. This assay revealed a significantly decreased survival rate in the *rel1*Δ mutant compared with the wild-type under this condition (Fig. S4B). In addition, when cultured on this host-mimicking condition, a significantly decreased production of extracellular polysaccharide capsule was observed in the cell population of the *rel1*Δ mutant, compared with those observed in wild-type and the complemental strain (Fig. S4C). In contrast, no detectable difference was observed between these strains in melanin production (Fig. S4D). These results indicated an important role of Rel1 in adapting to host-relevant conditions and the production of the capsule.

It was recently reported that a ribosome-associated factor, MSP-8, is involved in antifungal drug resistance in the model filamentous fungus *Neurospora crassa* (34). As Rel1 functions as a potential 60S ribosome biogenesis factor, we thus investigated whether Rel1 is involved in modulating susceptibility to the commonly used antifungal drugs in combating cryptococcal infection. To this end, a similar spotting assay was performed with the *REL1* disruption, complementation, and wild-type strains on a YPD plate supplied with the following three antifungal drugs: fluconazole (Flu), amphotericin B (AmB), and 5’-flucytosine (5-FC). As shown in Fig. 5B, the *rel1*Δ mutant exhibited a remarkable reduction in resistance to 5-FC and a moderate decrease in resistance to AmB. In contrast, dramatically enhanced resistance to fluconazole was observed in this mutant compared to the wild-type and complemented strains. The increased resistance to fluconazole of the *rel1*Δ mutant was also confirmed by growth curve assay on liquid YPD medium supplied with different concentrations of fluconazole (Fig. 5C). In addition, 10–15 µg/mL of fluconazole almost completely suppressed the growth of the wild-type strain in liquid YPD medium, even when the incubation time was extended to 9 days. In contrast, the *rel1*Δ mutant exhibited consistent growth under these fluconazole treatment conditions (Fig. 5C). The remarkable resistance to fluconazole and sensitivity to SDS indicated that the membrane ergosterol content may be altered in the mutant. To test this possibility, we examined the expression of the key ergosterol synthesis pathway genes *ERG2*, *ERG3*, *ERG11,* and *ERG25*. Unexpectedly, the expression of these *ERG* genes was significantly downregulated in the *rel1*Δ mutant upon fluconazole treatment compared with that observed in wild-type and the complemental strain (Fig. 5D). This result implied that the fluconazole-resistant phenotype of the *rel1*Δ mutant may not result from ergosterol biosynthesis. Collectively, these results indicated a critical role of Rel1 in modulating anti-fungal drug susceptibility in *C. neoformans*.

## DISCUSSION

Sexual reproduction is ubiquitous across eukaryotic species, acknowledged as a critical driving force for environmental adaptation, survival, and evolution (35). In the human fungal pathogen *C. neoformans*, extensive meiotic recombination occurs during both unisexual and bisexual reproduction (14, 15), which plays a crucial role in facilitating ecological niche outspreading, infection, and drug-resistance evolution (8, 36, 37). Cryptococcal sexual reproduction is a precisely regulated process that responds to mating induction signals, such as carbon and nitrogen starvation, dehydration, and pheromones (4). These signals trigger a series of sequential morphological developmental events, including yeast-hyphae transition, hyphal extension, basidial differentiation, meiosis, and the final sporulation (4). Despite extensive research conducted over the past two decades to identify the key signaling pathways and transcriptional regulators involved in Cryptococcus sexual development, the critical regulator(s) beyond the transcriptional machinery remain incompletely understood. In this study, we identified a gene, *CNB00600,* encoding a C2H2 zinc finger protein named Rel1, which was dynamically upregulated under mating-inducing conditions (Fig. 1A and B). Phylogenetic analysis and sequence alignment assay reveal that Rel1 is highly homologous to ScRei1, a well-characterized 60S ribosome biogenesis factor in *S. cerevisiae* (Fig. 1C and D). ScRei1 has been shown to play a critical role in sustaining cellular growth under low-temperature conditions in both yeast and plants (21, 26). Similarly, deletion of the Rel1 encoding gene also led to a low temperature-sensitive growth phenotype (Fig. 2A and B) as observed in the *rei1* disruption mutant in *S. cerevisiae* (21). Complementation of ScRei1 into the *rel1*Δ mutant restored growth to wild-type levels at 25°C, further confirming the functional conservation between Rel1 and ScRei1. However, the differences in subcellular localization and phenotypic characteristics also highlight the divergent functions of these two C2H2 zinc finger proteins. ScRei1 is consistently localized to the cytoplasm throughout the cell cycle (21). In contrast, Rel1 exhibits dual localization patterns: it localizes to the cytoplasm under mating repressing condition (YPD) but exhibits both cytoplasmic and nuclear localization in the hyphae cells under mating-inducing condition (V8) (Fig. 1E). Similarly, it has been reported that the ScRei1 homolog in the entomopathogenic fungus Beauveria bassiana, BbRei1, also shows cytoplasmic and nuclear localization (27), suggesting a broader subcellular localization diversity of Rei1-like proteins beyond what has been observed in *S. cerevisiae*. Furthermore, the failure of ScRei1 complementation in compensating for the growth defect of the *rel1*Δ mutant at 16°C also indicates the divergent function between these two proteins.

As the *REL1* gene is significantly upregulated in response to mating stimulation, its involvement in sexual reproduction was systematically investigated. Intriguingly, disruption of the *REL1* gene not only dramatically compromised yeast-hyphae morphogenesis but also blocked the later sexual development events, including basidial differentiation, meiosis, and sporulation (Fig. 3). Complementation with a wild-type copy of the *REL1* gene completely restored its sexual development defect. As Rel1 is a potential 60S ribosome biogenesis factor, these results indicate a strong association between the ribosome biogenesis pathway and sexual reproduction in *C. neoformans*. This hypothesis is supported by our RNA-seq data that multiple GO terms involved in ribosome biogenesis and rRNA processing are significantly enriched in the upregulated genes in wild-type strain under mating-inducing condition (V8 medium) compared with that on mating-repressing condition (YPD medium) (Fig. 4B). In addition to ribosome biogenesis, the enriched Gene Ontology (GO) terms among the up-regulated genes also include multiple metabolic processes, such as transmembrane transport, polysaccharide metabolic process, carbohydrate metabolic process, and response to zinc ion (Fig. 4B). The upregulation of these biological processes may reflect an increased demand for nutrients and energy to initiate sexual development. Furthermore, the significantly enriched biological processes also include sexual reproduction and pheromone-dependent signal transduction, which are consistent with the cellular responses observed under mating-inducing conditions. In contrast, many of these upregulated biological processes are downregulated when *REL1* is deleted. These processes include transmembrane transport, carbohydrate metabolic process, polysaccharide metabolic process, small molecular catabolic process, cellular amino acid catabolic process, response to zinc ion, sexual reproduction, and pheromone-dependent signal transduction. Notably, the critical role of Rel1 in facilitating the activation of the known gene sets involved in the entire cryptococcal sexual development is exhibited by heatmap analysis (Fig. 4C) and confirmed by RT-PCR assay (Fig. 4E) and cell fusion analysis (Fig. 4F). Taken together, this genome-wide transcriptional profile analysis indicates that Rel1 is crucial for promoting the activation of cryptococcal mating and sexual reproduction.

In *S. cerevisiae*, ScRei1 plays a critical role in the dissociation of the pre-60S maturation factors Arx1, Alb1, and Tif6 from the late cytoplasmic pre-60S particle, and their recycling into the nucleus (22, 23). Consequently, deletion of ScRei1 results in constant cytoplasmic localization of these late ribosome biogenesis factors and impairs the subsequent maturation of the 80S ribosome (23). Phenotypically, ScRei1 deletion causes significant growth retardation at low temperature. Deletion of Arx1 or Alb1 in the *rei1*Δ mutant background restores the nucleus import of Tfi6 and compensates the cold-sensitive phenotype of the rei1Δ strain (23), suggesting a close association between the ribosome biogenesis process and cold stress adaptation/resistance. However, homologs of Arx1 and Alb1 are absent in *C. neoformans* and any other basidiomycetes, indicating divergent evolution of the late ribosome biogenesis processes between basidiomycetes and yeast. Similarly, the conserved role of Rei1 homologs in maintaining cellular growth at low temperature has also been reported in the fungal insect pathogen *Beauveria bassiana* and the plant *Arabidopsis thaliana* (26, 27). In comparison, we demonstrate a much more diverse function of the Rei1 homolog, Rel1, in the human fungal pathogen *C. neoformans*. In addition to maintaining growth under low temperature conditions and safeguarding sexual development, Rel1 also showed a pleiotropic role in resistance to anti-fungal drugs. Disruption of *rel1* resulted in significant sensitivity to and 5-fluorocytosine but exhibited increased resistance to fluconazole (Fig. 5B and C). However, the increased resistance to fluconazole is not correlated with expression of ergosterol biogenesis pathway genes, as the mRNA level of the four core *ERG* genes is significantly decreased in the *rel1*Δ mutant compared with WT and the complemental strain (Fig. 5D). This result implies that Rel1 may modulate fluconazole susceptibility through an unknown mechanism beyond the ergosterol biogenesis pathway. The involvement of ribosome-associated factor in antifungal drug resistance has also been reported recently (34). In the model filamentous fungus *Neurospora crassa*, a DEAD/DEAH-box helicase MSP-8, which interacts with the ribosomal subunit proteins and participates in protein translation, is identified to be involved in multidrug resistance through experimental evolution screening. Disruption of MSP-8, or suppressing protein translation with translation inhibitor treatment, leads to resistance to multiple antifungal drugs (34). This result implies that mutation with ribosome biogenesis-related genes may help develop antifungal drug resistance at the cost of fitness, which could be a common mechanism for achieving antifungal drug resistance across fungal species. In summary, the present study unveiled that Rel1, a conserved C2H2 zinc finger protein, functions as a pleiotropic regulatory factor involved not only in maintaining growth under low temperature but also in sexual development and antifungal susceptibility in *C. neoformans*, providing a paradigm of the conserved ribosome biogenesis pathway in orchestrating diverse physiological processes in a ubiquitous human fungal pathogen.

## MATERIALS AND METHODS

### Strains and culture conditions

*Cryptococcus neoformans var. neoformans* (serotype D) reference strain XL280 was used throughout this work as a parental strain for gene knockout and control strains for phenotypic assay. All the strains constructed in this study are listed in Table S3. *Cryptococcus* strains were routinely cultured on YPD medium (1% yeast extract, 2% peptone, 2% glucose, and 2% agar) at 30°C and supplemented with 100 µg/mL nourseothricin (Solarbio, Beijing, China), 100 µg/mL G418 (Transgen, Beijing, China), or 200 µg/mL hygromycin (Solarbio, Beijing, China) for selection when necessary. Unisexual/bisexual filamentation and sporulation assay were performed on V8 juice agar medium (0.5 g/L KH_2_PO_4_, 5% V8 juice [wt/vol], and 4% Bacto agar, pH 7.0) at a 25°C incubator in the dark.

### Strain and plasmid construction

Previously published TRACE system was used in this research for gene knockout, complementation, and overexpression (38, 39). Briefly, to knock out the *REL1* gene in XL280, around 1 kb of the 5′ and the 3′ flanking fragments of the *REL1* open reading frame (ORF) were amplified from XL280 genomic DNA and fused with a G418 resistance marker to generate the gene knock-out cassette via overlapping PCR. Similarly, the guide RNA fragment target *REL1* ORF was constructed through fusing the U6 promoter, 20 bp target sequence, and the sgRNA scaffold together. The Cas9 encoding construct was amplified from the plasmid pXL1-CAS9-HYG with the universal primers M13F/M13R. The deletion cassette, CAS9 fragment, and sgRNA fragment were mixed and co-transformed into XL280 via electroporation, and the transformants were selected on a G418-resistant plate. The deletion strain was confirmed through the genetic stability test, followed by genomic diagnostic PCR confirmation.

For the construction of the *REL1* complementation strain, the expression fragment encompassing the native promoter and ORF of the *REL1* gene was amplified from XL280 genomic DNA. This fragment was then ligated into plasmid pXL1-HYG via NotI and PacI restriction enzymes digestion and seamless cloning to generate the *REL1* complementation plasmid P*_REL1_-REL1-HYG*. To construct the N-terminal mNeonGreen-labeled Rel1 recombinant strain, the *REL1* ORF was amplified from XL280 genomic DNA and subsequently ligated into the linearized plasmid pFZ1-HYG (FseI/PacI digested), which contains an mNeonGreen tag under the control of the *CTR4* promoter and a HYG selective marker, to generate the plasmid pFZ1-*REL1-HYG* (p*CTR4-mNeonGreen-REL1-HYG*). To construct the ScRei1 overexpression strains, the ORFs of these two genes were amplified from the genomic DNA of the *S. cerevisiae* Y187 strain and cloned into the pFZ1-HYG plasmid via ApaI/PacI digestion, followed by seamless cloning to generate the final plasmid p*CTR4*-Sc*REI1*-HYG. The complementation and overexpression cassettes with HYG selective marker were amplified from the resulting plasmids using M13F/M13R primers and then co-transformed with a SH3 sgRNA and CAS9 construct into the *rel1*Δ strain via electroporation, respectively. Transformants were selected on G418-containing plates and confirmed by genomic diagnostic PCR following a genetic stability test. All the primers used for strain construction are listed in Table S4.

### Rel1 ortholog identification and phylogenetic analysis

The NCBI BLASTp program was used to identify the potential Rel1 orthologs across Ascomycetes and Basidiomycetes. The domain architecture of the representative Rel1 homologs from the indicated fungal species was predicted by using the NCBI CDD program (https://www.ncbi.nlm.nih.gov/Structure/cdd/wrpsb.cgi) and visualized with IBS software (http://ibs.biocuckoo.org/download.php). A phylogenetic tree of the Rel1 orthologs was constructed using the neighbor-joining method implemented in MEGA7 software.

### Cell cycle analysis

For cell cycle analysis, the *rel1* deletion, complementation, and XL280 strains were cultured on YPD plates at 30°C, 25°C, and 16°C for 24 h, respectively. Cells were then collected and washed twice with sterile water prior to cell cycle analysis, which was performed by flow cytometry as previously described (40). Briefly, these cells were fixed with one milliliter of 70% ethanol at 4 for overnight and then washed once with one milliliter of cold NS buffer (10 mM Tris-HCl pH 7.5, 250 mM Sucrose, 1 mM EDTA, 1 mM MgCl_2_, 0.1 mM CaCl_2_, 0.1 mM ZnCl_2_, 0.4 mM PMSF, 7 mM β-mercaptoethanol) and stained with 5 µL of propidium iodide (0.5 mg/mL) (P8080, Solarbio) and 10 µL of RNase A (20 mg/mL) (GE101-01, Transgen) in 180 µL NS buffer at room temperature for 3 h. After that, the stained cells were diluted 100 times with 50 mM TE buffer (pH = 8.0) and sonicated for 1 min. Flow cytometry was performed with 10,000 cells for each sample and analyzed on the FL1 channel on a CytoFLEX S flow cytometer (Beckman Coulter) at Jiangxi Institute of Respiratory Diseases. FlowJo (v10.0) software was used for cell cycle data analysis.

### Filamentation, basidial maturation, and sporulation assay

Filamentation and sporulation assays were performed as previously described (41). For the unisexual filamentation and sporulation assay, cells cultured overnight on YPD plates were harvested, washed three times with sterile water, and then adjusted to an OD600 of 0.2 or 0.01 for the following experiment. For filamentation initiation assay, the diluted cells (OD600 = 0.01) were dropped onto the V8 plate and incubated at 25°C in the dark for 24 h. Morphology of the mini colonies at the early mating stage (24) was observed and photographed under an optical microscope (BX43, Olympus). Filamentation initiation efficiency was determined by calculating the ratio of the filamentous mini-colonies compared with the total number of colonies. At least 50 mini-colonies were calculated for each strain. For hyphal development, cells with OD600 = 0.2 were dropped onto V8 medium and cultured under identical conditions. The morphology of the colony was observed and captured by a stereo microscope (S9D, Leica) after 3 days of incubation on the V8 agar plate, and basidiospore chain formations were observed and photographed under an optical microscope (BX43, Olympus) after 2 weeks of incubation.

The Basidium Maturation Score (BMS) assay was conducted according to a previously published method (28). Briefly, hyphal cells from the edge of each mating colony were carefully scraped and resuspended in 1× PBS and then examined using a glass slide under an optical microscope (Zeiss AXIO lab.A1). The BMS value was calculated based on the ratio of basidial diameter to its connected hyphae diameter, which was measured under an optical microscope (Zeiss AXIO Lab.A1) and analyzed using software Zen 2011. For the BMS statistical analysis, 80 hyphae from each strain were randomly selected and measured.

### Microscopical fluorescence assay

To investigate the subcellular localization of Rel1, the P*_CTR4_-mNeonGreen-REL1* recombinant strain was pre-cultured on liquid YPD medium overnight and then washed and spotted onto a YPD plate for 24 h and on V8 medium for 3 days, respectively. The yeast cells and hyphae cells were carefully scraped from the colony edge of the YPD and V8 cultures, respectively. The cells were then resuspended in a diluted Hoechst 33342 (10 μg/mL) (HY-15559, MCE) solution for nuclear staining. Subsequently, the fluorescence signals were observed under a fluorescent microscope (DMi8, Leica).

To examine the impact of Rel1 disruption on Dmc1 expression, the strains expressing Dmc1-mCherry under control of its native promoter were cultured on V8 medium for 1 week. The hyphae cells were carefully scraped from the colony edge and gently resuspended in sterile 1× PBS buffer. In addition, 10 µL of these hyphae cells was subsequently dropped onto a glass slide for fluorescent microscopic examination. All images were taken with a fluorescent microscope (DMi8, Leica), and the fluorescence intensity of Dmc1-mCherry from 65 hyphae apices of each strain was quantified using Las X software.

### RNA extraction, reverse transcription, and RT-qPCR

For RNA extraction, the overnight pre-cultured *rel1* deletion, complement strain, and XL280 were collected, washed twice with sterile water, and adjusted to OD600 = 0.5. These cell suspensions were spotted onto V8 medium and incubated at 25°C in the dark for 24 h. Following incubation, these cells were harvested and washed with cold 1× PBS twice and stored at −80°C freezer before RNA extraction. Total RNA extraction was performed by using a commercial Ultrapure RNA kit (CWBIO, CW0581S, China) according to the manufacturer’s protocol. The HiScript III RT SuperMix kit (plus gDNA wiper, R323, Vazyme, Nanjing, China) was used for reverse transcription following genomic DNA digestion. Quantitative real-time PCR was performed with an ABI real-time PCR detection system (QuantStudio Dx) and using the reagent ChamQ Universal SYBR qPCR Master Mix (Q711, Vazyme, Nanjing, China) according to the manufacturer’s protocol. Gene expression levels were normalized to an endogenous gene, *TEF1,* and analyzed by using the relative quantitation/comparative threshold cycle (ΔΔCt) method. Two biological replicates and three technical replicates were performed for each sample. All the primers used in RT-qPCR are listed in Table S4.

### RNA-seq

For transcriptome analysis, overnight pre-cultured wild-type and *rel1*Δ mutant strains were harvested, washed, and spotted onto V8 agar medium. The cultures were then grown at 25°C for 24 h. As a control, an additional group was set up by spotting wild-type strain cells onto a YPD plate and incubating at 30°C for 24 h. Three biological replicates were prepared for each strain under each condition. Cells were rapidly collected, washed twice with cold 1× PBS, snap-frozen in liquid nitrogen, and stored at -80°C. Total RNA was extracted using the Ultrapure RNA Kit (CW0581S, CWBIO, China) according to the manufacturer’s protocol. Subsequently, genomic DNA was removed from the total RNA using the TURBO DNA-free Kit (AM1907, Thermo Fisher, USA) prior to library construction. RNA purity and concentration were assessed using the NanoDrop 2000 spectrophotometer (Thermo Scientific, USA), and RNA integrity was evaluated using the Agilent 2100 Bioanalyzer (Agilent Technologies, Santa Clara, CA, USA). Libraries were constructed using the VAHTS Universal V6 RNA-seq Library Prep Kit (NR604, Vazyme, Nanjing, China) following the manufacturer’s instructions. Transcriptome sequencing and analysis were performed by OE Biotech Co., Ltd. (Shanghai, China). Sequencing was conducted on the Illumina Novaseq 6000 platform, and 150 bp paired-end reads were generated. Clean reads were mapped to the reference genome JEC21α using HISAT2 (42). FPKM values of each gene were calculated, and the read counts of each gene were obtained by HTSeq-count (43). Differential expression analysis was carried out using DESeq2 (44), with a threshold of q-value < 0.05 and│log2(Fold change)│> 1.0 set for significantly differentially expressed genes (DEGs). Venn diagram, GO enrichment analysis, and heatmap were conducted and visualized using the online bioinformatics tools (https://cloud.oebiotech.cn/task/) provided by OE Biotech Co., Ltd. (Shanghai, China).

### Growth, stress sensitivity, and antifungal susceptibility assays

Strains were pre-cultured overnight in liquid YPD medium at 30°C with shaking and subsequently collected, washed twice with sterile water, adjusted to a consistent cell density (OD600 = 2.0), and serially diluted tenfold from 10^0^ to 10^−4^. For the growth assay at various temperatures, 3 µL aliquots of each dilution were spotted onto YPD plates and incubated at 16°C, 25°C, 30°C, and 37°C for 1–3 days, with daily photographic documentation. To assess cold stress tolerance at 4°C, an extra YPD plate with identically spotted cells was incubated at 4°C and photographed at 10- and 30-day incubation.

For growth curve analysis in liquid YPD medium, each strain was inoculated into 100 mL of YPD medium in a flask with an initial cell density to OD_600_ = 0.1 and incubated with shaking at 220 rpm at 16°C and 30°C, respectively. Growth curves were generated by measuring the OD600 of each culture every 12 h using a spectrophotometer (model T6, Persee, Beijing).

For stress tolerance and antifungal drug susceptibility assay, the serially diluted cells were spotted onto YPD agar medium supplemented with the following chemical agents: oxidative stress (1 mM tBOOH, 3 mM H_2_O_2_), osmotic stress (1.5 M NaCl, 1.5 M sorbitol), DNA damage stress (0.01% MMS, 50 mM HU), ER stress (15 mM DTT), cell wall/membrane-destabilizing stress (1% Congo Red, 0.005% SDS), and antifungal drugs (0.2 µg/mL amphotericin B, 15 µg/mL fluconazole, and 200 µg/mL 5-flucytosine). These plates were incubated at 30°C and photographed after 3 days.

## Data Availability

RNA-Seq data are available via the Gene Expression Omnibus (GEO) database on NCBI (https://www.ncbi.nlm.nih.gov/geo/) with identifier GSE296246. Other data are available upon request.

## SUPPLEMENTAL MATERIAL

Supplemental figures aem.01460-25-s0001.docx Figures S1 to S5.

Table S1 aem.01460-25-s0002.xlsx DEGs in WT strain on V8 medium compared to that on YPD.

Table S2 aem.01460-25-s0003.xlsx DEGs in *rel1*Δ mutant on V8 compared to WT on V8.

Table S3 aem.01460-25-s0004.docx Strains and plasmids used in this study.

Table S4 aem.01460-25-s0005.xlsx Primers used in this study.

ASM does not own the copyrights to Supplemental Material that may be linked to, or accessed through, an article. The authors have granted ASM a non-exclusive, world-wide license to publish the Supplemental Material files. Please contact the corresponding author directly for reuse.
