# Supplementary material for: A conserved C2H2 zinc finger protein, Rel1, links ribosome biogenesis to sexual development and antifungal susceptibility in a ubiquitous human fungal pathogen
Source: Appl Environ Microbiol. 2025 Nov 25;91(12):e01460-25. doi: 10.1128/aem.01460-25 (PMC12724378; doi:10.1128/aem.01460-25)
Supplement: Supplemental figures — Figures S1 to S5. [file aem.01460-25-s0001.docx]

**Supplemental figures**

**A conserved C2H2 zinc finger protein, Rel1, links ribosome biogenesis to sexual development and antifungal susceptibility in a ubiquitous human fungal pathogen**

Lili Yan^1,2,3†^, Man Chen^1,4†^, Yuanli Liu^5^, Yan Wang^6^, Pengjie Hu^7^, Yanli Cao^8^, Ye Huang^1,2,3^, Guojian Liao^6^, Xiuyun Tian^7^, Xinping Xu^1,2,3^, Fanglin Zheng^1,2,3*^

^1^Jiangxi Provincial Key Laboratory of Respiratory Diseases, Jiangxi Institute of Respiratory Diseases, The Department of Respiratory and Critical Care Medicine, The First Affiliated Hospital, Jiangxi Medical College, Nanchang University, Nanchang, Jiangxi 330006, China.

^2^Jiangxi Clinical Research Center for Respiratory Diseases, Nanchang, Jiangxi 330006, China

^3^Jiangxi Hospital of China-Japan Friendship Hospital, Nanchang, Jiangxi 330006, China

^4^Division of Nephrology, Renmin Hospital of Wuhan University, Wuhan, China

^5^Department of Critical Care Medicine, The First Affiliated Hospital of Gannan Medical College, Ganzhou, Jiangxi, China

^6^State Key Laboratory of Mycology, Institute of Microbiology, Chinese Academy of Sciences, Beijing 100101, P. R. China

^7^College of Pharmaceutical Sciences, Southwest University, Chongqing 400700, China

^8^School of Basic Medical Sciences, Jiangxi Medical College, Nanchang University, Nanchang, 330006, China

^†^Lili Yan and Man Chen contributed equally to this work.

*Correspondence: fanglin.zheng@ncu.edu.cn (F.Z.)

**Figure S1**


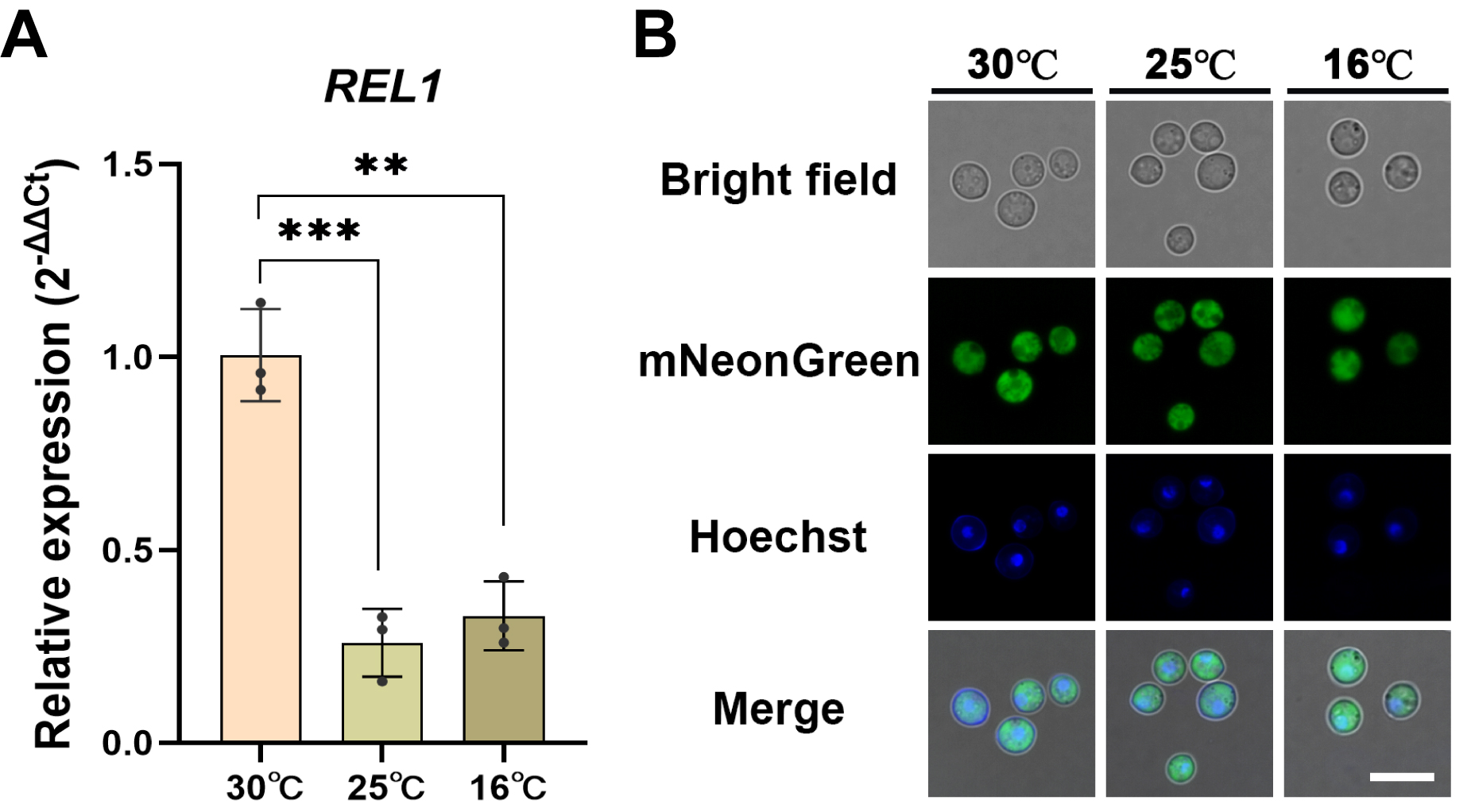


**Fig. S1 *REL1* gene is downregulated in response to cold stress, and its encoded protein consistently localizes to the cytoplasm across various temperature conditions.** (**A**) Transcriptional dynamics of *REL1* in response to temperature shifts from 30°C to 25°C and 16°C. Error bar indicated mean ± SD from three independent biological replications of each culture conditions. Statistical significance was determined by two-tailed Student’s t-test (***, p<0.001; **, p<0.01). (**B**) Subcellular localization of mNeonGreen-Rel1 under normal and low temperature culture conditions. Hoechst 33342 was used for nuclear staining. Scale bar, 10 μm.

**Figure S2**


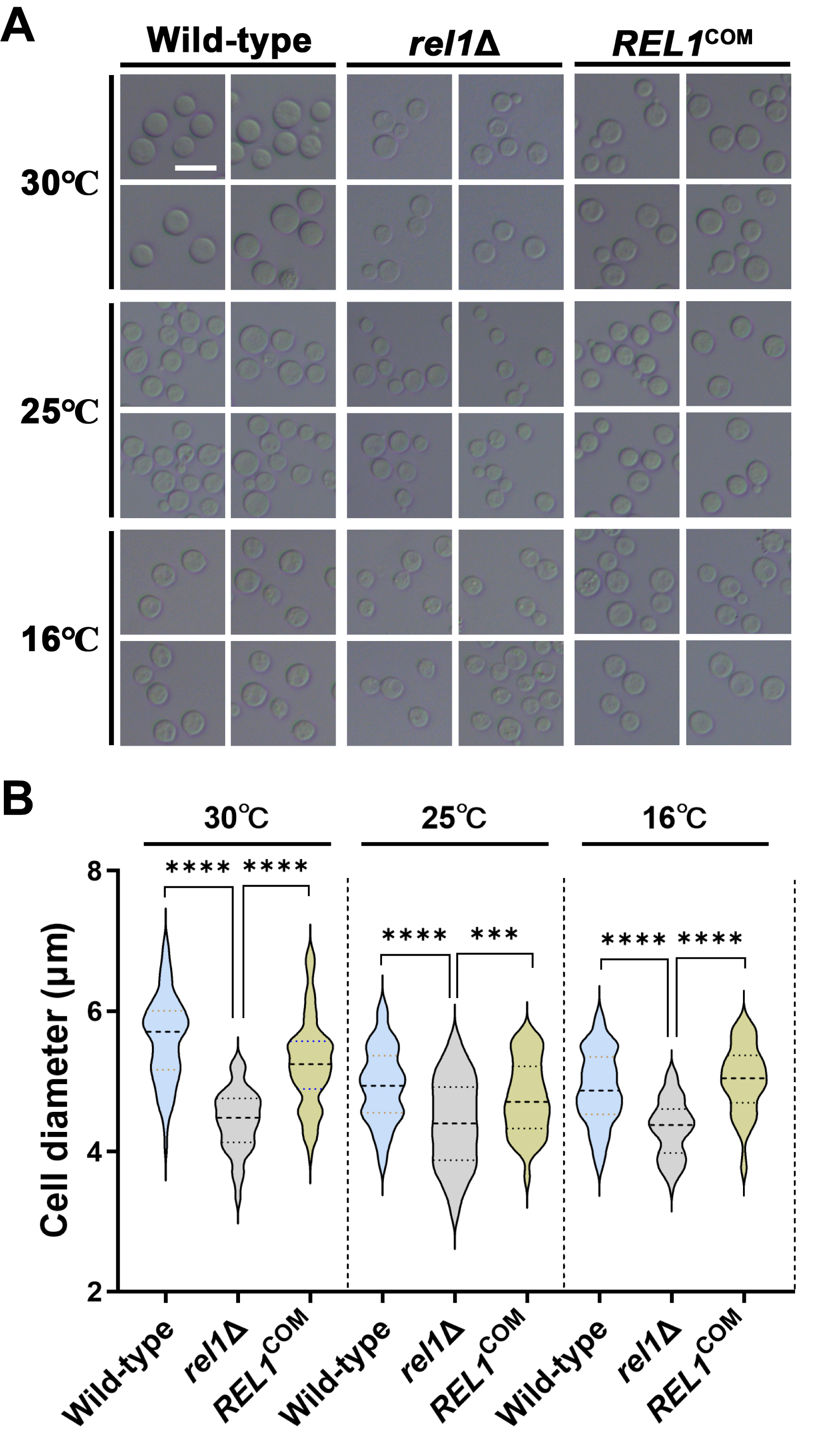


**Fig. S2 Cellular morphology and cell size analysis between *REL1* deletion, complementation, and the wild-type strains under the indicated temperature culture conditions.** (**A**) Optical microscopic observation of the cellular morphology of the indicated yeast strains cultured on YPD medium at temperatures of 30°C, 25°C, and 16°C respectively. Scale bar, 10 μm. (**B**) Violin plot analysis shows the disruption of cell size among the indicated strains under normal and low temperature culture conditions. Eighty cells from each strain under each culture conditions were randomly selected for cell diameter measurement. Statistical significance was determined by two-tailed Student’s t-test (***, p<0.001; ****, p<0.0001).

**Figure S3**


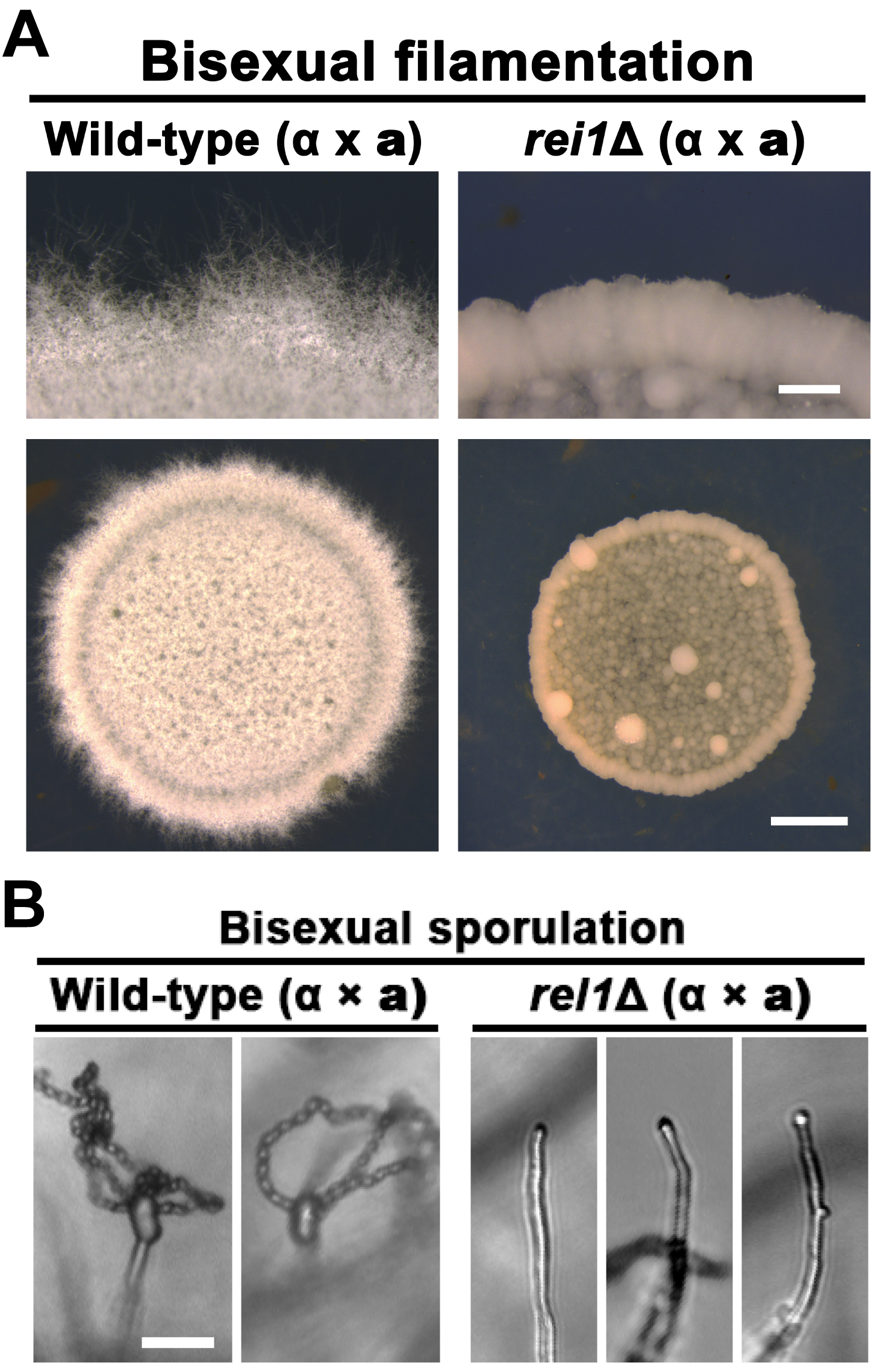


**Fig. S3 Rel1 plays a critical role in completing bisexual reproduction.** (A) The impact of *REL1* deletion on hyphal development during bisexual reproduction. Images were captured under a stereoscope after five days incubation on V8 agar medium. Scale bars represent 200 µm for upper panel and 1 mm for lower panel respectively. (B) Sporulation analysis of the wild-type cross and *rel1*Δ mutant cross under mating condition on V8 medium. The images of hyphae tips of each strain were captured after two weeks of incubation on V8 medium. Scale bar, 20 μm.

**Figure S4**


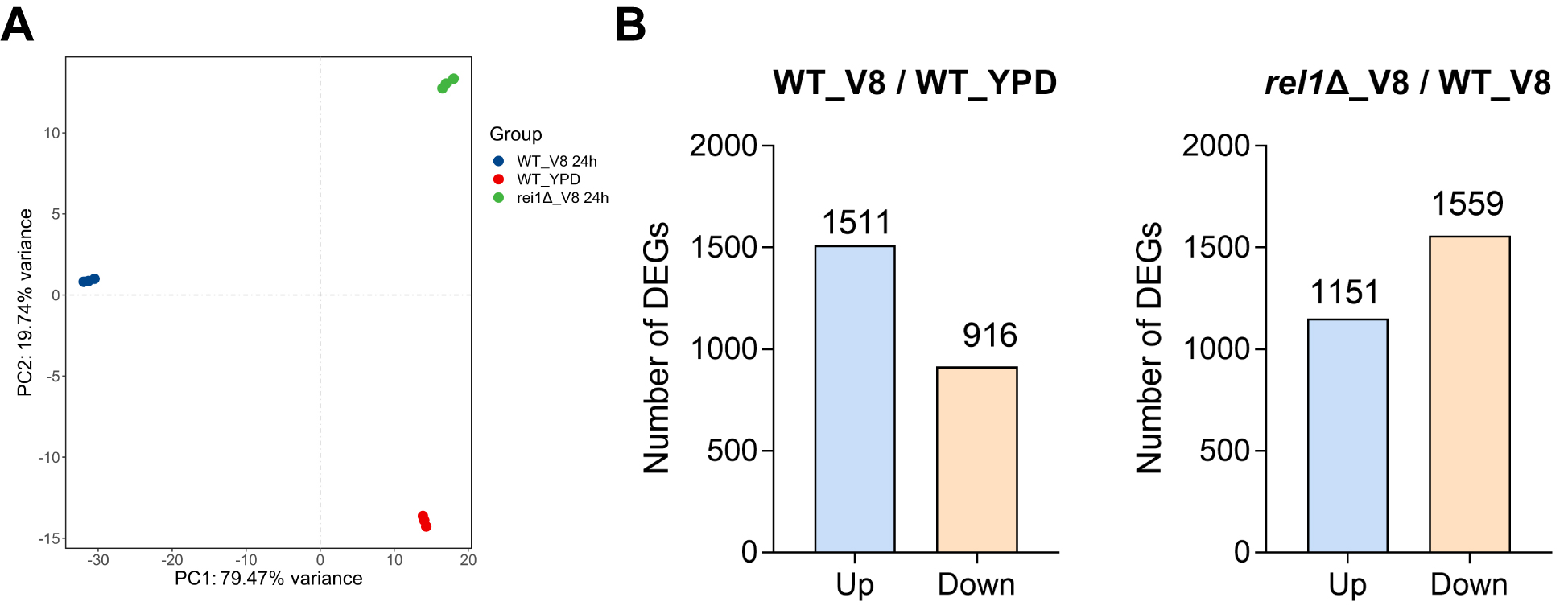


**Fig. S4 PCA assay (A) of the RNA-Seq samples and DEGs number (B) of the indicated comparative groups.**

**Figure S5**


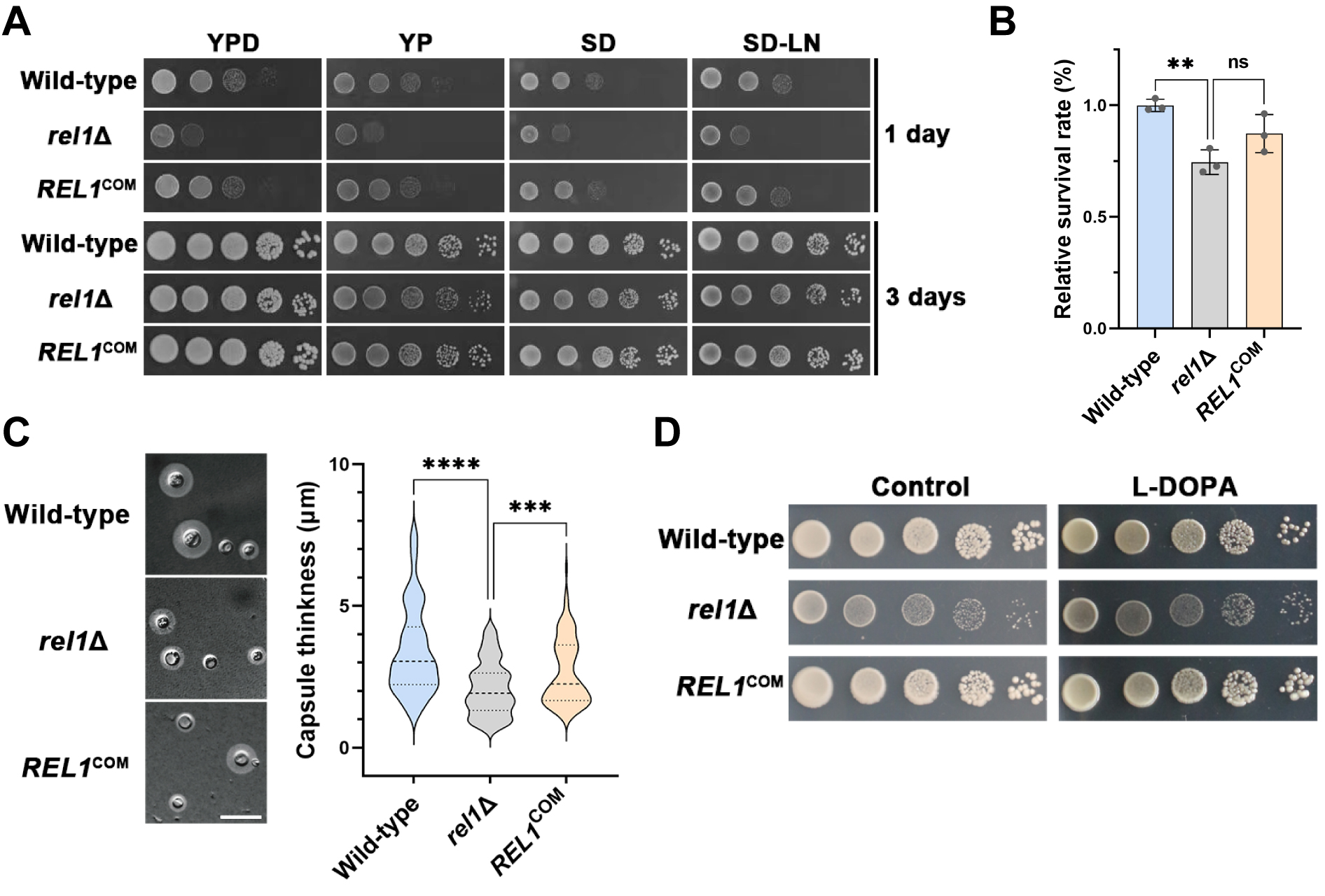


**Fig. S5 Phenotypic assay of *rel1*Δ mutant under nutrition limitation and host-mimicking conditions.** (**A**) Growth assay of the indicated strains under carbon and nitrogen source limitation conditions. The strains were 10 times seriously diluted and spotted onto YPD, YP (without glucose and any other carbon source), SD (0.17% YNB + 0.5% (NH₄)₂SO₄) and SD-LN (low-nitrogen SD medium containing 0.05% (NH₄)₂SO₄), and then incubated at 30℃ for the indicated time period. (B) Survival assay of the indicated strains following exposure to host-mimicking culture condition on RPMI medium with 5% CO₂ at 37°C for 24 hours. The survival rate was determined by calculating the ratio of the CFU number emerged on YPD medium to the total cell number counted using a hemocytometer under microscope, following incubation under above culture condition. (C) Capsule production assay of the indicated strains on RPMI medium with 5% CO₂ at 37°C for five days. One hundred cells of each strain were randomly selected for capsule thickness measurement. Scale bar, 10 μm. Statistical significance was determined by two-tailed Student’s t-test (***, p<0.001; ****, p<0.0001). (D) Melanin production assay of the indicated strains on L-DOPA medium.
