# Supplementary material for: A conserved C2H2 zinc finger protein, Rel1, links ribosome biogenesis to sexual development and antifungal susceptibility in a ubiquitous human fungal pathogen
Source: Appl Environ Microbiol. 2025 Nov 25;91(12):e01460-25. doi: 10.1128/aem.01460-25 (PMC12724378; doi:10.1128/aem.01460-25)
Supplement: Table S3 — Strains and plasmids used in this study. [file aem.01460-25-s0004.docx]

**Table S3. Strains and plasmids used in this study.**

**Strains**

| **Strain Name** | **Background** | | Genotype |  |
| --- | --- | --- | --- | --- |
| **XL280** | Wild type | | MATα, serotype D wild type strain |  |
| **JEC20a** | Wild type | | MAT**a**, serotype D wild type strain |  |
| **XP294** | XL280 | | MATα, *rel1*::NEO |  |
| **XP378** | XL280 | | MATα, *mat2*::NAT |  |
| **XP389** | XL280 | | MATα, P*_DMC1_*-*DMC1*-mCherry-HYG |  |
| **XP410** | XL280 | | MATα, SH3::NEO |  |
| **XP413** | JEC20**a** | | MAT**a**, SH3::HYG |  |
| **LY6** | XL280 | | MATα, *rel1*::NEO, P*_REI1_*-*REI1*-HYG |  |
| **LY71** | XL280 | | MATα, *rel1*::NEO, P*_CTR4_*-mNeoGreen-*REI1*-HYG |  |
| **LY106** | XL280 | | MATα, *rel1*::NEO, P*_DMC1_*-*DMC1*-mCherry-HYG |  |
| **LY121** | XL280 | | MATα, *rel1*::NEO, P*_DMC1_*-*DMC1*-mCherry-HYG, P*_CTR4_*-*REI1*-NAT |  |
| **LY128** | XL280 | | MATα, *rel1*::NEO, P*_CTR4_* - *ScREI1*-HYG |  |
| **LY132** | XL280 | | MATα, P*_REI1_* -EGFP-NEO |  |
| Plasmids | | | |  |
| **Plasmid name** | | **Backbone** | **Annotation** |  |
| pXL1-HYG | | pXL1 | Overexpression plasmid with pGPD1-*Fse*I-*Pac*I-tGPD1 construct and HYG selective marker |  |
| pXL1-CAS9-HYG | | pXL1 | CAS9 expression plasmid |  |
| pREL1-*REL1*-HYG | | pXL1-HYG | *REL1* complementation plasmid |  |
| pFZ1-HYG | | pFZ1 | Overexpression plasmid with pCTR4-*Apa*I-mNeonGreen-*Fse*I-*Pac*I-tGPD1 construct and HYG selective marker |  |
| pFZ1-*REL1*-HYG | | pFZ1-HYG | P*_CTR4_*-mNeoGreen-*REL1* expression plasmid under control of *CTR4* promoter |  |
| pCTR4-Sc*REI1*-HYG | | pFZ1-HYG | *ScREI1* complementation plasmid under control of *CTR4* promoter |  |
